# Altered Expression of the m6A Methyltransferase METTL3 in Alzheimer’s Disease

**DOI:** 10.1523/ENEURO.0125-20.2020

**Published:** 2020-09-08

**Authors:** He Huang, Judith Camats-Perna, Rodrigo Medeiros, Victor Anggono, Jocelyn Widagdo

**Affiliations:** 1 Clem Jones Centre for Ageing Dementia Research, Queensland Brain Institute, The University of Queensland, Brisbane, Queensland 4072, Australia; 2 Institute for Memory Impairments and Neurological Disorders, University of California, Irvine, Irvine, CA 92697

**Keywords:** Alzheimer’s disease, epitranscriptomic, METTL3, N6-methyladenosine, neurodegenerative disorders, RNA methylation

## Abstract

Cognitive impairment in Alzheimer’s disease (AD) is associated with dysregulation of the RNA and protein expression profiles in the brain. Recent studies have highlighted the importance of RNA post-transcriptional regulation (epitranscriptomics) in higher order brain functions. Specifically, *N*6-methyladenosine (m6A), which controls RNA stability, splicing, translation and trafficking, plays an important role in learning and memory. This raises the question of whether m6A signaling is perturbed in AD. To address this, we investigated the expression profile of known m6A-regulatory genes using a public RNA-seq dataset and identified a subset of genes which were significantly dysregulated in the human AD brain. Among these, genes encoding the m6A methyltransferase, *METTL3*, and a member of the m6A methyltransferase complex (MACOM), *RBM15B*, were downregulated and upregulated in the hippocampus, respectively. These findings were validated at the protein level using an independent cohort of postmortem human brain samples. Unexpectedly, we observed an accumulation of methyltransferase-like 3 (METTL3), but not RBM15B, in the insoluble fractions, which positively correlated with the levels of insoluble Tau protein in the postmortem human AD samples. Aberrant expression and distribution of METTL3 in the hippocampus of the AD brain may therefore represent an epitranscriptomic mechanism underlying the altered gene expression patterns associated with disease pathogenesis.

## Significance Statement

*N*6-methyladenosine (m6A), the most prevalent internal RNA modification in eukaryotes, controls various aspects of RNA metabolism. m6A plays an important role in learning and memory; however, whether m6A signaling is perturbed in Alzheimer’s disease (AD) remains unknown. Here we report a decrease in the messenger RNA (mRNA) expression that encodes the key m6A methyltransferase enzyme, *METTL3*, in the postmortem hippocampal tissues of AD patients. We also identified a striking alteration in the methyltransferase-like 3 (METTL3) protein expression, including enhanced insolubility and immunoreactivity in the AD hippocampus. This suggests that perturbation of m6A signaling may present a novel cellular mechanism underpinning dysregulation of gene expression associated with AD pathophysiology.

## Introduction

Alzheimer’s disease (AD) is a chronic neurodegenerative disorder that is characterized by a progressive decline in higher cognitive function and memory loss (Tarawneh and Holtzman, 2012). Accumulation of insoluble neurotoxic aggregates, including amyloid-β (Aβ) plaques and intracellular Tau neurofibrillary tangles, represents a major pathologic hallmark of AD that leads to synaptic dysfunction and ultimately neuronal death and dementia (Polanco et al., 2018). Changes in gene and protein expression profiles underpin dysfunction in many fundamental cellular processes during disease pathogenesis in AD (Neueder, 2019). One of the mechanisms underlying AD-related changes in gene expression involves perturbations of the epigenome via disease-specific changes in chromatin structure and/or transcription programs (Sanchez-Mut and Graff, 2015). These include alterations in DNA methylation (De Jager et al., 2014; Lunnon et al., 2014) and histone modifications (Marzi et al., 2018; Nativio et al., 2018; Klein et al., 2019).

In recent years, chemical modifications in RNA have emerged as important mechanisms for the control of gene expression and protein translation (Saletore et al., 2012; Zhao et al., 2017). *N*6-methyladenosine (m6A), the most abundant and reversible post-transcriptional modification on eukaryotic messenger RNAs (mRNAs), is a versatile regulator of mRNA stability, splicing, localization, and translation rate (Schwartz, 2016; Zhao et al., 2017). In mammals, covalent attachment of a methyl group to an adenosine at the *N*6 position is catalyzed by the multiprotein m6A methyltransferase complex (MACOM) that comprises the catalytic subunit methyltransferase-like 3 (METTL3) which forms a heterodimer with METTL14 (Liu et al., 2014). The precise localization of the METTL3/14 complex is determined by the Wilm’s tumor 1-associating protein (WTAP; Ping et al., 2014). Additional co-factors in the MACOM which facilitate its anchoring and targeting in nuclear speckles and U-rich regions adjacent to m6A sites in mRNAs include Vir-like m6A methyltransferase associated (VIRMA), zinc finger CCCH-type containing 13 (ZC3H13), Casitas B-lineage lymphoma-transforming sequence-like protein 1 (CBLL1; also known as HAKAI), RNA binding motif protein 15 (RBM15), and RBM15B (Huang et al., 2020b). Recognition of m6A by m6A readers, specific RNA binding proteins that reside in either the nucleus or the cytoplasm dictates the processing outcome of the methylated transcripts (Yang et al., 2018; Shi et al., 2019). Removal of the methyl groups is mediated by two distinct m6A demethylating enzymes, namely the fat mass and obesity-associated (FTO) protein and AlkB homolog 5 (ALKBH5; Jia et al., 2011; Zheng et al., 2013).

The widespread presence of m6A in the neuronal transcriptome underpins its numerous functional roles throughout development and adult brain function (Widagdo and Anggono, 2018; Flamand and Meyer, 2019; Livneh et al., 2020). Accumulating evidence has demonstrated essential roles of m6A signaling in learning and memory by facilitating the translation of plasticity-related genes in the mouse brain (Widagdo et al., 2016; Li et al., 2017; Walters et al., 2017; Koranda et al., 2018; Shi et al., 2018; Zhang et al., 2018). Given that learning and memory impairments are clinical hallmarks of AD, we posited that dysregulation of m6A signaling may be associated with the pathophysiology of AD. By examining the expression profiles of m6A regulatory genes in postmortem human AD brains, we report the aberrant expression of MACOM proteins, including METTL3 and RBM15B, in the hippocampus, indicating a potential perturbation in m6A signaling that may contribute to neuronal dysfunction in AD.

## Materials and Methods

### DNA constructs and antibodies

The plasmid encoding Mettl3 was generated by amplifying total mouse brain cDNA using the forward primer 5′-ATAGTCGACGATGTCGGACACGTGGAGCTCT-3′ and the reverse primer 5′- TTGCGGCCGCGTGCGTCTATAAATTCTTAGGTT-3′. The PCR products were digested with SalI and NotI (New England Biolabs) and ligated into the cut pRK5-myc vector. Plasmids encoding V5-tagged Tau (wild-type and P301L mutant) were generously provided by Prof. Jürgen Götz (Li and Götz, 2017).

Antibodies were purchased from commercial sources as follows: anti-METTL3 (ab195352, Abcam), anti-RBM15B (2249-1-AP, Proteintech), anti-METTL14 (HPA038002, Sigma), anti-Tau (MN1000, Thermo Scientific), anti-V5 (V8137, Sigma or clone7/4, Biolegend), anti-myc (clone 9E10, Bio-Rad Laboratories or clone A-14, Santa Cruz Biotechnology), anti-MAP2 (ab92434, Abcam), or anti-β-actin (sc-47 778, Santa Cruz Biotechnology).

### *In silico* transcriptomic database analyses

To investigate the expression of m6A-related genes in the postmortem AD brain of male and female subjects, we analyzed a publicly available transcriptomic dataset from the Aging, Dementia and TBI Study (http://aging.brain-map.org) by the Allen Institute for Brain Science (Miller et al., 2017). The demographic and clinical information of donors associated with this study is summarized in Table 1. Adjusted normalized fragments per kilobase of transcript per million (FPKM) mapped reads were used to assess differential gene expression.

**Table 1:** Demographic summary of donors associated with the Allen Institute’s Aging, Dementia and TBI Study

	Hippocampus		Parietal cortex		Temporal cortex	
Group	Non-Dementia	Dementia	Non-Dementia	Dementia	Non-Dementia	Dementia
Sample size	29	21	27	21	28	23
Age (years)	88.2 ± 6.4	91.0 ± 6.0	88.4 ± 6.6	90.7 ± 6.1	88.2 ± 6.5	90.1 ± 5.8
Female percentage	48.3%	38.1%	51.9%	28.6%	46.4%	30.4%
APOE4 alleles	7.1%	38.9%^**^	11.5%	36.8%^*^	11.1%	38.1%^*^

* *p *<* *0.05.

** *p *<* *0.01, χ^2^ test for gender and APOE4 composition among groups. An unpaired *t* test was used for age analysis.

### Human postmortem brain tissue

Postmortem brain tissues from cognitively normal and AD individuals (males and females) were obtained from the neuropathology core of the Alzheimer’s Disease Research Center at the University of California, Irvine (for subject details, see Table 2 and Extended Data Table 2-1). The protocols for obtaining these tissues complied with all guidelines with special respect for donor identity confidentiality and informed consent and were approved by the University of California, Irvine Institutional Review Board. Paraformaldehyde-fixed and frozen hippocampal tissues were provided for this study. Experiments were performed according to the approval granted by the University of Queensland Human Research Ethics Committee (#2017000490).

**Table 2:** Demographic summary of donors from the Alzheimer’s Disease Research left at University of California, Irvine

Group	Control	AD	*p* value
Sample size	19	26	-
Age (years)	90.3 ± 4.4	88.6 ± 6.1	0.31
Female percentage	52.6%	53.8%	0.94
APOE4 alleles	31.6%	46.2%	0.32

χ^2^ test for gender and APOE4 composition among groups. An unpaired *t* test was used for age analysis.

10.1523/ENEURO.0125-20.2020.t2-1 Extended Data Table 2-1 Human brain samples from normal and Alzheimer’s disease patients analyzed in this study. Download Table 2-1, DOCX file.

### Protein extraction and Western blot analysis

Frozen hippocampal tissues were ground into powder in liquid nitrogen, transferred into a Precellys homogenization tube (Bertin Technologies) and lysed in ice-cold T-PER extraction buffer (Thermo Scientific) containing protease and phosphatase inhibitors as described previously (Zumkehr et al., 2015). Briefly, tissue lysates were cleared by ultracentrifugation at 100,000 × *g* for 60 min at 4°C to obtain the soluble fractions. The resulting pellet was resuspended with 70% formic acid and again homogenized, followed by another ultracentrifugation step at 100,000 × *g* for 60 min at 4°C. The supernatant was collected as the insoluble fraction. The protein concentration was determined using the Bradford assay (Bio-Rad Laboratories). After normalizing the protein concentration, samples were mixed with 3× SDS sample buffer and denatured at 95°C for 10 min. Equal amounts of protein were loaded on 10% acrylamide gels and transferred to polyvinylidene fluoride membranes. The membranes were then blocked, washed and incubated with the specific primary antibodies overnight. After washing and incubation with horseradish peroxidase-conjugated secondary antibodies, detection with enhanced chemiluminescence was performed using an Odyssey Fc imaging system (Li-COR). Image Studio software (Li-COR) was used to acquire images and perform densitometry analyses. Quantification of the Aβ levels in the T-PER insoluble fractions was performed using ELISA as described previously (Medeiros et al., 2014).

### Co-immunoprecipitation assays

Co-immunoprecipitation assays were conducted according a previously published protocol (Anggono et al., 2013). Briefly, HEK293T cells were cultured in DMEM containing 10% fetal bovine serum, 50 U/ml penicillin, and 50 μg/ml streptomycin at 37°C in a humidified 5% CO_2_ tissue culture incubator. Cells were transfected using the calcium phosphate precipitation method and lysed 48 h later with ice-cold cell lysis buffer (1% Triton X-100, 1 mm EDTA, 1 mm EGTA, 50 mm NaF, and 5 mm Na-pyrophosphate in PBS) containing protease inhibitors. Cell lysates were cleared at 17,000 × *g* for 20 min at 4°C and incubated with antibodies coupled to protein A-Sepharose overnight at 4°C. Beads were washed extensively with ice-cold cell lysis buffer and eluted with 2× SDS sample buffer. Bound proteins were resolved by SDS-PAGE followed by Western blot analysis.

### Immunohistochemistry

Paraformaldehyde-fixed hippocampal tissues were sliced into 20-μm sections using a sliding microtome (SM1020R, Leica). The immunohistochemistry procedure was performed as previously described (Medeiros et al., 2014), including an antigen retrieval step (Tris/EDTA pH 9.0 at 97°C for 10 min). Staining with anti-METTL3 antibody (ab195352, Abcam, 1:50 dilution) was performed overnight at 4°C, followed by detection with biotinylated horse anti-rabbit IgG (Vector Laboratories), avidin-biotin complex (Vector Laboratories), and diaminobenzidine (DAB) staining. Stained hippocampal sections were imaged with a Zeiss Axio Imager Z2 microscope equipped with a 20× objective and a Metafer slide scanning platform. Quantification of the optical density (OD) of images was performed with Fiji software (ImageJ, NIH). Briefly, color deconvolution of the images was achieved using the set OD vectors for DAB and hematoxylin (H DAB). The OD value of the image presenting DAB was calculated using the formula OD = log(max intensity/mean intensity). Selected regions from CA1, CA2, CA3, and the dentate gyrus (DG) were measured separately for each section.

### Immunofluorescence staining

Sections were first blocked with 5% normal serum, 2% BSA, and 0.1% Tween 20 in TBS at room temperature. Primary antibody incubation was performed overnight at 4°C. Finally, sections were rinsed and incubated for 1 h with Alexa Fluor-conjugated secondary antibodies (Invitrogen) at room temperature. Images were collected with a 63× oil-immersion objective on a Zeiss LSM510 confocal microscope.

### Statistical analysis

Data collected in this study were tested for normality using the Kolmogorov–Smirnov test (Prism 7, GraphPad). Estimation-based confidence intervals (CIs) were calculated using the DABEST estimation statistics package with the permutation *t* test in Python 3.7.6 (Ho et al., 2019). For each estimation plot, (1) raw data were plotted on the upper axes (or left side) with their mean ± SD indicated by the vertical gapped lines; and (2) their effect size was plotted on the lower or right floating axes with the mean difference and 95%CIs indicated by dot and the ends of the vertical error bar, respectively. For each permutation *t* test, 5000 bootstrap samples were taken. The CI was bias-corrected and accelerated, and the resulting *p* value is the likelihood of observing the effect size, if the null hypothesis of zero difference is true. The statistical tests applied in each figure are summarized in Table 3. We also performed unpaired (two-tailed) Student’s *t* tests for the RNA-seq data analysis (Table 4). A χ^2^ test was used to compare the gender and APOE4 composition between the AD and control groups (Tables 1, 2).

**Table 3:** Statistical summary of the mRNA and protein changes associated with AD brain tissues

Figure	Data	Data structure (normality test)	Type of test	Power	*p* value
1*A*	METTL3 (HIP)	Yes	Permutation *t* test	[95%CI –0.75,–0.03]	0.049
	METTL3 (PCX)	Yes	Permutation *t* test	[95%CI –0.63,0.12]	0.204
	METTL3 (TCX)	Yes	Permutation *t* test	[95%CI –0.63,0.15]	0.220
1*B*	RBM15B (HIP)	Yes	Permutation *t* test	[95%CI 0.06,0.83]	0.040
	RBM15B (PCX)	Yes	Permutation *t* test	[95%CI 0.09,0.81]	0.032
	RBM15B (TCX)	Yes	Permutation *t* test	[95%CI 0.14,0.83]	0.012
2*B*	Soluble METTL3	No	Permutation *t* test	[95%CI –0.75,–0.05]	0.030
2*C*	Soluble RBM15B	No	Permutation *t* test	[95%CI 0.11,0.85]	0.036
2*D*	Soluble METTL14	Yes	Permutation *t* test	[95%CI –0.74,0.02]	0.074
2*E*	Soluble tau	No	Permutation *t* test	[95%CI –0.33,0.08]	0.219
3*B*	Insoluble METTL3	No	Permutation *t* test	[95%CI 1.27,5.31]	0.012
3*C*	Insoluble tau	No	Permutation *t* test	[95%CI 5.53,18.76]	0.003
4*D*	IHC (CA1)	Yes	Permutation *t* test	[95%CI 0.48,5.15]	0.015
	IHC (CA2)	No	Permutation *t* test	[95%CI 0.42,1.87]	0.014
	IHC (CA3)	Yes	Permutation *t* test	[95%CI 0.25,2.11]	0.025
	IHC (DG)	Yes	Permutation *t* test	[95%CI 0.36,1.70]	0.007

HIP = hippocampus; PCX = parietal cortex; TCX = temporal cortex; IHC = immunohistochemistry.

**Table 4:** Summary data of m6A-related gene profiles in control versus AD subjects

		HIP			PCX			TCX		
			*p* value			*p* value			*p* value	
Writer	*METTL3*	↓	0.0487	*		n.s.			n.s.	
	*METTL14*		n.s.			n.s.			n.s.	
MACOM	*CBLL1*		n.s.			n.s.			n.s.	
	*RBM15*		n.s.			n.s.			n.s.	
	*RBM15B*	↑	0.0388	*	↑	0.0337	*	↑	0.0141	*
	*ZC3H13*		n.s.			n.s.			n.s.	
	*VIRMA*		n.s.			n.s.			n.s.	
	*WTAP*		n.s.			n.s.			n.s.	
Eraser	*FTO*		n.s.			n.s.			n.s.	
	*ALKBH5*		n.s.			n.s.		↑	0.0143	*
Reader	*EIF3A*		n.s.			n.s.			n.s.	
	*ELAVL3*		n.s.			n.s.		↑	0.0337	*
	*ELAVL4*		n.s.			n.s.			n.s.	
	*YTHDF1*		n.s.			n.s.			n.s.	
	*YTHDF2*		n.s.			n.s.			n.s.	
	*YTHDF3*		n.s.			n.s.			n.s.	
	*HNRNPA2B1*		n.s.		↑	0.0142	*		n.s.	
	*FMR1*		n.s.			n.s.			n.s.	
	*IGF2BP1*		n.s.			n.s.			n.s.	
	*IGF2BP2*		n.s.			n.s.			n.s.	
	*IGF2BP3*		n.s.			n.s.			n.s.	
	*YTHDC1*		n.s.			n.s.			n.s.	
	*YTHDC2*		n.s.			n.s.			n.s.	

HIP = hippocampus; PCX = parietal cortex; TCX = temporal cortex; MACOM = m6A-methyltransferase associated complex.

↑ and ↓ indicate an increase and decrease, respectively, in the gene expression pattern in AD compared with control subjects.

n.s. = not significant; *p <* *0.05; unpaired *t* test.

## Results

**Figure 1.**
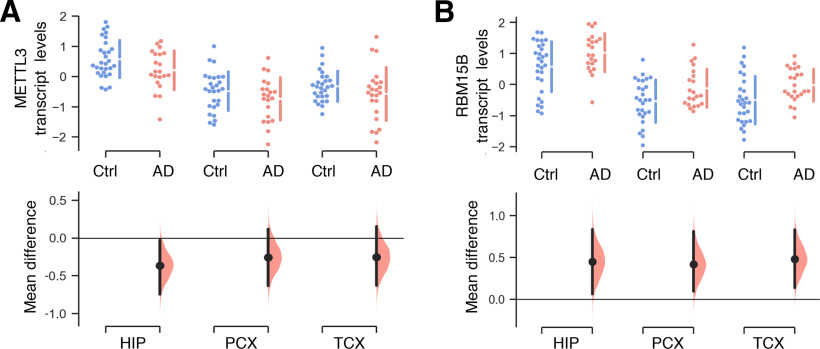
Altered expression of METTL3 and RBM15B transcripts in the AD brain. *In silico* analyses of METTL3 (***A***) and RBM15B (***B***) mRNA expression in various postmortem human brain tissues from AD patients (pink dots) and control subjects (blue dots) from the Allen Institute for Brain Science transcriptomic database. Raw data are shown on the upper axes. Estimation plots below display the mean differences between the AD and control groups. METTL3 (HIP, M_diff_ = −0.37 [95%CI −0.75,−0.03], *p* = 0.049; PCX, M_diff_ = −0.26 [95%CI −0.63,0.12], *p* = 0.204; TCX, M_diff_ = −0.25 [95%CI −0.63,0.15], *p* = 0.220). RBM15B (HIP, M_diff_ = 0.45 [95%CI 0.06,0.83], *p* = 0.040; PCX, M_diff_ = 0.42 [95%CI 0.09,0.81], *p* = 0.032; TCX, M_diff_ = 0.48 [95%CI 0.13,0.83], *p* = 0.012). HIP, hippocampus (control, *n* = 29; AD, *n* = 21); PCX, parietal cortex (control, *n* = 27; AD, *n* = 21); TCX, temporal cortex (control, *n* = 28; AD, *n* = 23).

To investigate the relative expression of m6A regulators in the postmortem AD brain, we first analyzed a publicly available transcriptomic dataset from the Allen Institute’s Aging, Dementia and TBI Study (Miller et al., 2017). A total of 23 genes with known functions in the m6A signaling pathway, including m6A writers, erasers, and readers (Huang et al., 2020b), were examined based on their levels of expression in three distinct brain regions (hippocampus, parietal cortex, and temporal cortex) of AD patients relative to healthy age-matched and sex-matched subjects (Table 1). Using unpaired (two-tailed) Student’s *t* tests, we identified a number of genes which were differentially expressed in the AD subjects relative to the control group as summarized in Table 4. Significantly dysregulated genes included *METTL3* (downregulated in the hippocampus), *RBM15B* (upregulated in all brain regions), *ALKBH5* and *ELVAL3* (upregulated in the temporal cortex), and *HNRNPA2B1* (upregulated in the parietal cortex). Interestingly, *METTL3* (Fig. 1*A*) and *RBM15B* (Fig. 1*B*), both of which are essential in m6A deposition, were the only two transcripts identified as being differentially expressed in the hippocampus, a key region of the brain for learning and memory.

**Figure 2.**
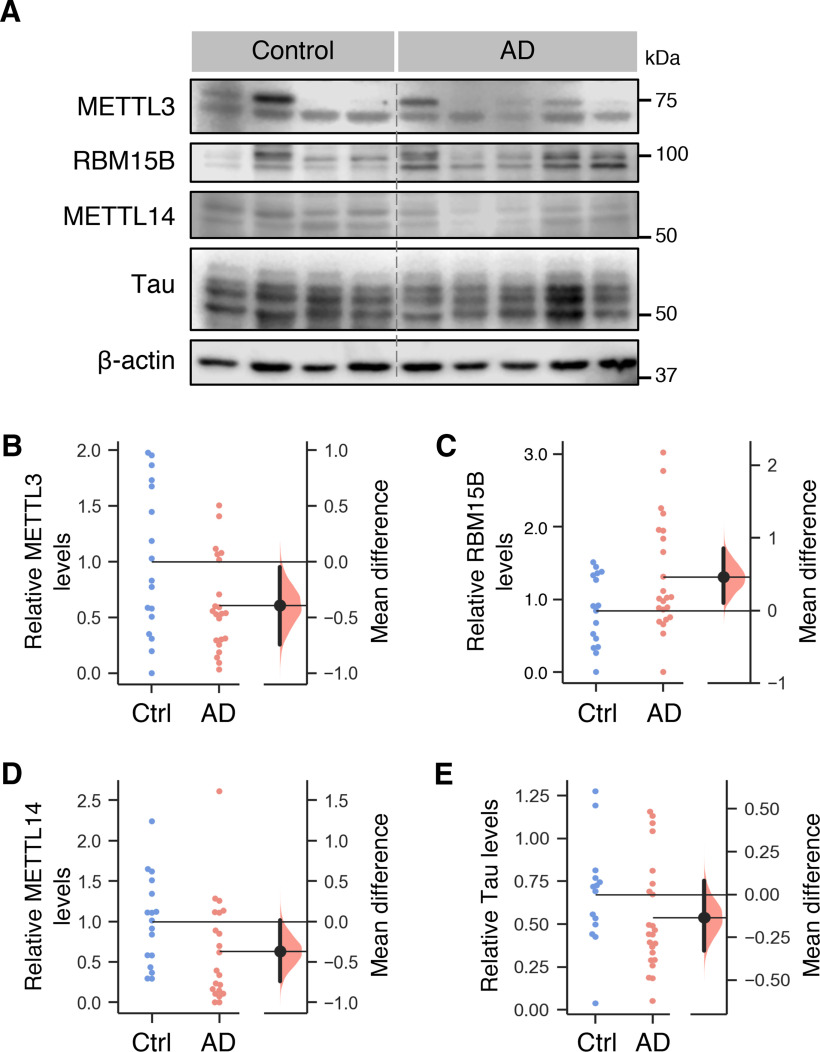
Analyses of METTL3, RBM15B, and METTL14 protein levels in the soluble fractions of postmortem AD hippocampal tissues. ***A***, Postmortem hippocampal tissue from AD patients and control subjects from the Alzheimer’s Disease Research Center of the University of California, Irvine, was lysed in T-PER buffer. Detergent-soluble fractions of the lysates were subjected to SDS-PAGE and Western blotting with specific antibodies against METTL3, RBM15B, METTL14, Tau, and β-actin. Representative blots are shown. Densitometry analyses of the blots for METTL3 (***B***), RBM15B (***C***), METTL14 (***D***), and Tau (***E***) after normalization with β-actin are presented as estimation plots. Raw data are plotted on the left (control, blue dots; AD, pink dots), and mean differences are shown on the right. METTL3 (M_diff_ = −0.39 [95%CI −0.75,−0.05], *p* =* *0.030), RBM15B (M_diff_ = 0.46 [95%CI 0.11,0.85], *p *=* *0.036), METTL14 (M_diff_ = −0.37 [95%CI −0.74,0.02], *p *=* *0.074) and Tau (M_diff_ = −0.13 [95%CI −0.33,0.08], *p *=* *0.219). AD (*n* =* *21–23), control (*n* =* *12–17).

To determine whether AD-associated changes in the m6A writer complex could be detected at the protein level, we obtained hippocampal tissues from an independent cohort of human postmortem AD and control subjects (Table 2; Extended Data Table 2-1). Western blot analysis revealed a significant downregulation in the level of detergent-soluble METTL3 (Fig. 2*A,B*), while the level of RBM15B was markedly increased in the AD subjects compared with the control group (Fig. 2*A*,*C*). These data were consistent with the changes observed at the transcript level (Fig. 1). No significant changes were observed in the levels of METTL14 (Fig. *2A*,*D*) and Tau proteins (Fig. 2*A*,*E*) in the soluble fractions of the AD samples.

**Figure 3.**
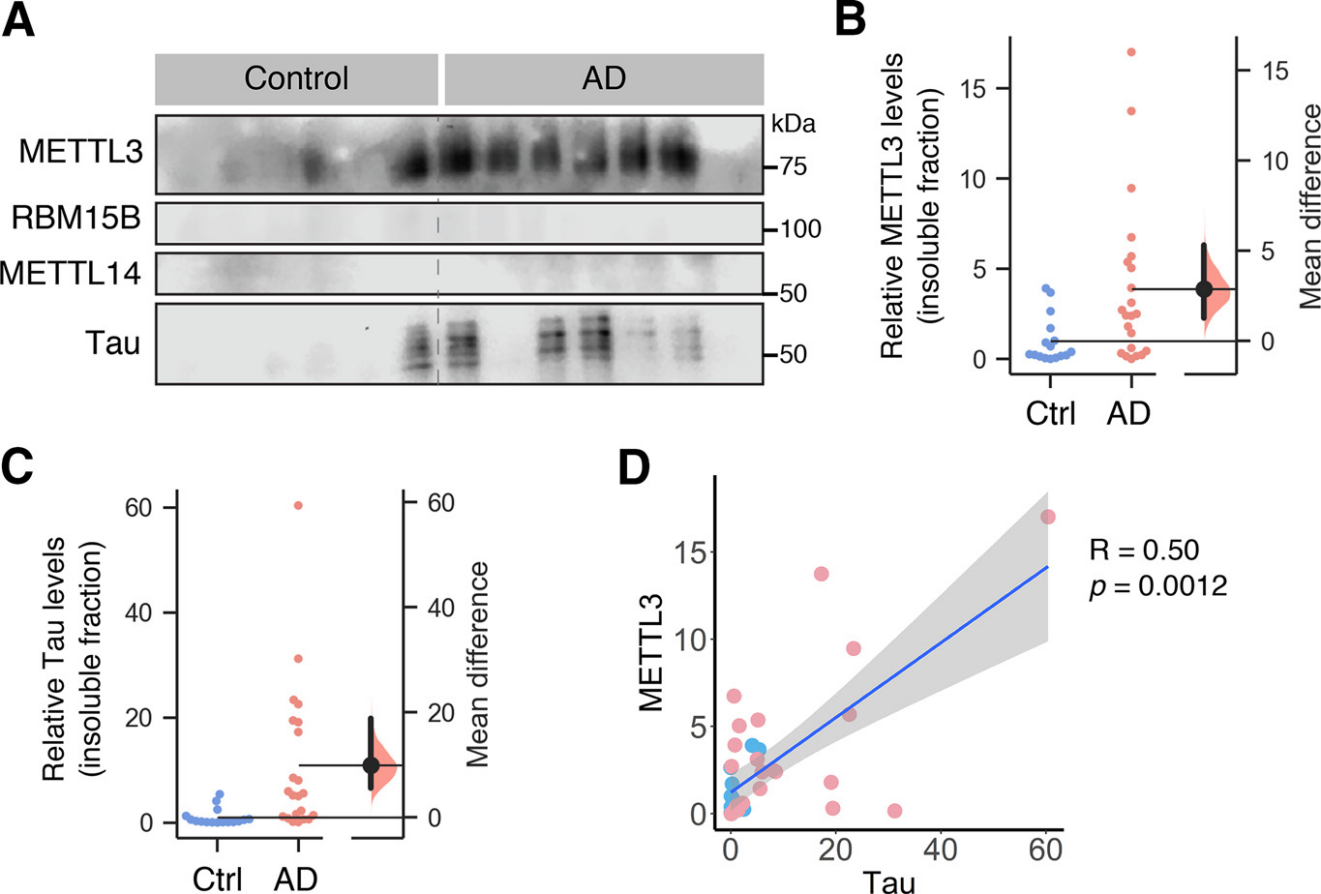
Elevated level of METTL3 protein in the insoluble fraction of human postmortem AD hippocampal tissues. ***A***, Representative Western blots of detergent-insoluble fractions prepared from postmortem hippocampal tissue from AD patients and control subjects, probed with specific antibodies against METTL3, RBM15B, METTL14, and Tau. Densitometry analyses of the blots for METTL3 (***B***) and Tau (***C***) are presented as estimation plots. Raw data are plotted on the left (control, blue dots; AD, pink dots), and mean differences are shown on the right. METTL3 (M_diff_ = 2.87 [95%CI 1.27,5.31], *p *=* *0.012) and Tau (M_diff_ = 9.92 [95%CI 5.53,18.76], *p *=* *0.003). AD (*n* =* *22), control (*n *=* *16). ***D***, Accumulation of METTL3 in the insoluble fraction of human hippocampal tissues positively correlates with Tau aggregates (control, blue dots; AD, pink dots; Spearman’s correlation coefficient = 0.50, *p *=* *0.0012).

Proteinopathy in AD is often associated with the aggregation of proteins such as Tau, which accumulate in the insoluble fraction. To examine whether any of the m6A writers underwent aberrant aggregation in the hippocampal tissues of AD subjects, we isolated the detergent-insoluble fractions derived from these samples. We did not detect the presence of RBM15B or METTL14 in the insoluble fractions of any of the control or AD samples (Fig. 3*A*). Surprisingly, however, we observed a significant increase in the level of METTL3 in the insoluble fractions derived from the hippocampal tissues of AD patients relative to the control group (Fig. 3*A*,*B*). As expected, the AD hippocampal tissues also contained a significantly greater accumulation of insoluble Tau protein compared with the control group (Fig. 3*A*,*C*). Interestingly, the level of METTL3 positively correlated with that of Tau in the insoluble fraction of these hippocampal lysates (Fig. 3*D*). However, the same analysis revealed no correlation between the level of insoluble METTL3 with the levels of Aβ40 or Aβ42 in the hippocampus (Extended Data Fig. 3-1).

10.1523/ENEURO.0125-20.2020.f3-1 Extended Data Figure 3-1 The levels of Aβ40 or Aβ42 do not correlate with METTL3 accumulation in the insoluble fractions. Relative levels of Aβ40 (***A***) or Aβ42 peptides (***B***) of control (*n *=* *11–12, blue dots) and AD (*n *=* *14–17, pink dots) as measured by ELISA. Data are presented as estimation plots. Accumulation of METTL3 in the insoluble fraction of human hippocampal tissues does not correlate with Aβ40 (***C***) or Aβ42 (***D***) levels. Spearman’s correlation coefficients and *p* values are shown on each graph. Download Figure 3-1, TIF file.

**Figure 4.**
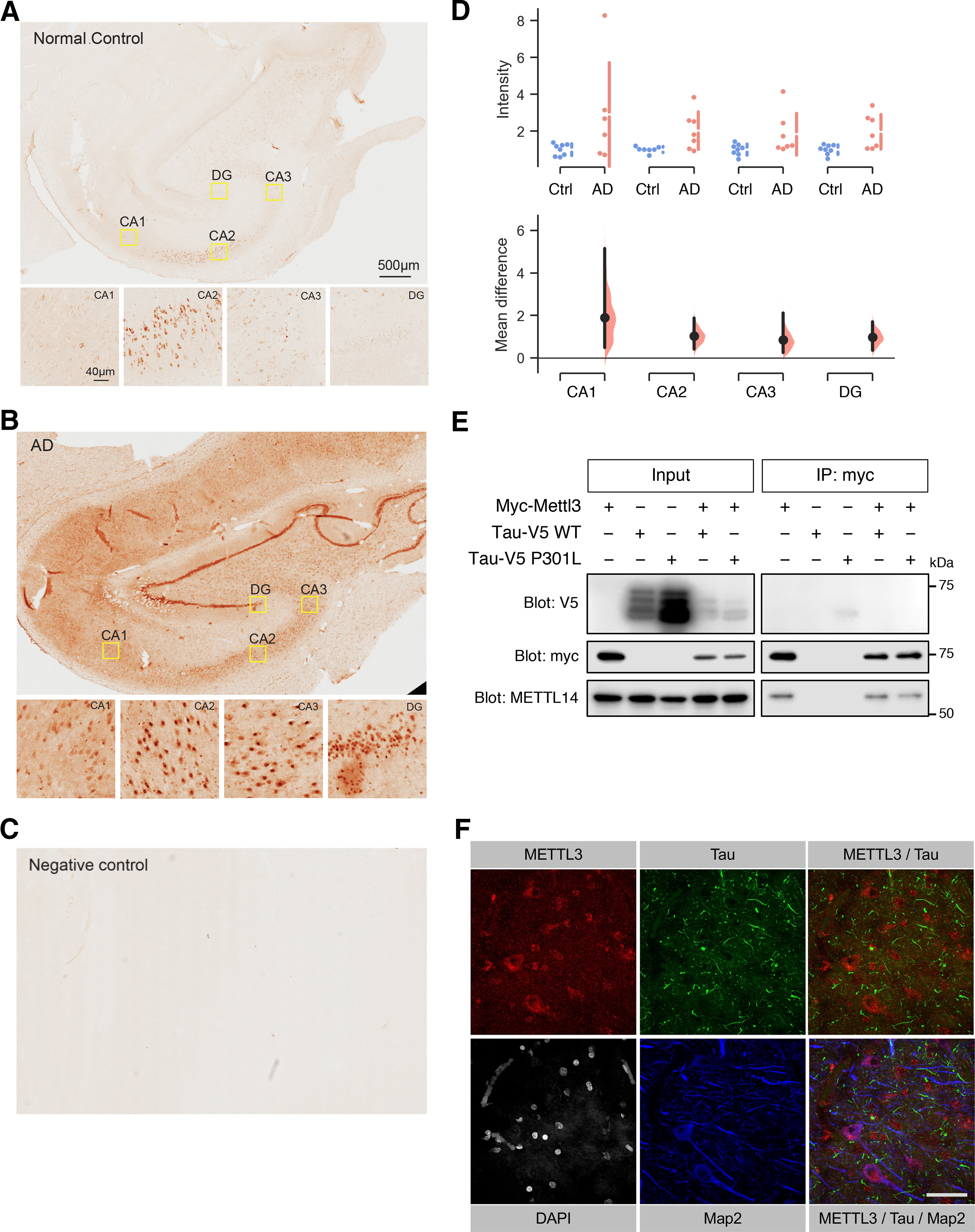
Analysis of METTL3 distribution and localization in postmortem human AD hippocampal tissue. Representative immunohistochemical staining of METTL3 in the human postmortem AD (***A***) and control hippocampal tissues (***B***). Magnified images of the selected regions are shown below. CA, cornu ammonis. Scale bar = 500 μm (low-power images) or 40 μm (high-power images). ***C***, Negative staining performed on AD hippocampal tissue using the same protocol without the primary antibody against METTL3. ***D***, Quantification of METTL3 OD measurement and mean differences in CA1 (M_diff_ = 1.90 [95%CI 0.48,5.15], *p *=* *0.015), CA2 (M_diff_ = 1.03 [95%CI 0.42,1.87], *p *=* *0.014), CA3 (M_diff_ = 0.84 [95%CI 0.25,2.11], *p *=* *0.025) and the DG (M_diff_ = 0.97 [95%CI 0.36,1.70], *p *=* *0.007). Data are presented as estimation plots, AD (*n *=* *6–7), control (*n *=* *7–8). ***E***, Mettl3 does not interact with Tau in cells. HEK293T cells were transfected with the indicated plasmids for 48 h, lysed and immunoprecipitated with anti-myc antibodies. Bound proteins were eluted and resolved by SDS-PAGE, and analyzed by Western blottings with specific antibodies against myc, V5 and METTL14. ***F***, Immunofluorescence staining revealed that METTL3 (red) and Tau (green) do not colocalize in AD hippocampal tissue. MAP2 and DAPI were used to stain for neuronal dendrites (blue) and nuclei (gray), respectively. Scale bar = 50 μm

To examine the distribution of METTL3, we performed immunohistochemical analyses on the paraformaldehyde-fixed hippocampal sections obtained from AD and control subjects (Fig. 4*A*,*B*). The lack of staining in an AD hippocampal section performed in the absence of anti-METTL3 confirmed the specificity of the immunohistochemistry procedure (Fig. 4*C*). In control tissues, relatively higher expression of METTL3 was apparent in the CA2 region (Fig. 4*A*). However, we observed more pronounced staining throughout the hippocampal formation, particularly in the DG, in the AD tissues (Fig. 4*B*). Quantitative OD measurement of the DAB staining confirmed significantly higher METTL3 immunoreactivity in all subregions of the hippocampal tissues of AD subjects relative to the controls (Fig. 4*D*).

The positive correlation in the levels of METTL3 and Tau in the insoluble fractions (Fig. 3) also prompted us to investigate the potential interaction between these two proteins. To address this, we co-transfected human Tau-V5, either wild-type or the AD-associated P301L mutant, with myc-Mettl3 plasmids in HEK293T cells and performed co-immunoprecipitation assays. As expected, endogenous METTL14 co-immunoprecipitated with myc-Mettl3, validating the specificity of the assay (Fig. 4*E*). However, co-immunoprecipitation using specific antibodies against myc revealed no interactions between Tau-V5 and myc-Mettl3 (Fig. 4*E*). Furthermore, immunofluorescence staining of METTL3 and Tau revealed a distinct subcellular localization of these two proteins in AD hippocampal tissues (Fig. 4*F*). Tau appeared as fibrillar structures, which is one of the pathologic hallmarks of AD. On the other hand, METTL3 was detected in the cell bodies of MAP2-positive neurons that did not possess intracellular neurofibrillary tangles. Collectively, these data suggest that although Tau pathology is a better predictor of deregulated m6A signaling than Aβ load, it is unlikely to play a causal role in altering METTL3 expression in the hippocampus of AD patients.

## Discussion

The m6A epitranscriptome adds to the complex regulation of RNA metabolism and function in neurons, rendering its essential roles in neuronal development, synaptic plasticity, cognition and the stress response (Engel and Chen, 2018; Huang and Lu, 2018; Widagdo and Anggono, 2018; Flamand and Meyer, 2019). The m6A methylome is dynamically regulated by sensory and learning experience in the hippocampus, striatum, and cortex of mice (Widagdo et al., 2016; Engel et al., 2018; Koranda et al., 2018; Zhang et al., 2018). Activity-dependent m6A deposition occurs on many transcripts of synaptic plasticity-related and immediate early genes (Widagdo et al., 2016; Shi et al., 2018; Zhang et al., 2018). Mice lacking the m6A writers Mettl3 or Mettl14 exhibit impairments in synaptic plasticity, learning, and memory consolidation (Koranda et al., 2018; Zhang et al., 2018). In contrast, increasing the abundance of m6A by knocking down the expression of the m6A demethylating enzyme, FTO, promotes memory consolidation in mice (Widagdo et al., 2016; Walters et al., 2017).

Alterations in RNA modifying enzymes have been widely implicated in human diseases, underscoring the importance of post-transcriptional control of RNA function in general physiology (Kadumuri and Janga, 2018). Dysregulation of global m6A abundance and aberrant expression of m6A writers, erasers, and readers are strongly associated with the pathophysiology of various cancer types and play critical roles in their initiation, progression, metastasis, and relapse (Li et al., 2019; Huang et al., 2020a). However, there has been little investigation of the epitranscriptomic changes that are associated with human neurodegenerative disorders. In genome-wide association studies, a gene variant in the m6A demethylase FTO has been reported to be a risk factor in AD (Ho et al., 2010; Keller et al., 2011), but the functional implication of this remains unclear. Here, we provide the first demonstration of dysregulated expression of the key m6A methyltransferase METTL3 in the human postmortem AD brain, indicating an impairment in m6A signaling in the pathophysiology of AD.

In mice, loss of METTL3 m6A methyltransferase function in the hippocampus has a negative impact on memory consolidation (Zhang et al., 2018). Downregulation of METTL3 mRNA and soluble protein levels in the AD hippocampus may therefore correlate with the memory dysfunction associated with this disease. The suppression of METTL3 function is further indicated by its increased insolubility, which positively correlates with the presence of Tau aggregates, but not with Aβ load or ApoE4 genotype (data not shown). This, accompanied by an elevated level and redistribution of METTL3 expression in AD hippocampal tissue, likely represents abnormal misfolding and/or aggregation of METTL3, perhaps resembling the frequent aggregation of RNA-binding proteins in neurodegenerative disorders (Arai et al., 2009; Conlon and Manley, 2017). Given that several m6A regulatory proteins are subjected to post-translational ubiquitination and SUMOylation (Zhang et al., 2015; Tai et al., 2017; Du et al., 2018; Zhu et al., 2018; Liu et al., 2020; Xu et al., 2020), it is conceivable that dysregulation of the ubiquitin-proteasome system may contribute to the aggregation of METTL3 in AD (Ihara et al., 2012; Zheng et al., 2016).

Interestingly, our findings are in stark contrast with a recent study that reported an increase in the mRNA and protein expression of Mettl3 in the APP/PS1 mouse model of AD (Han et al., 2020). A concomitant decrease in the levels of FTO transcript and protein was also observed, resulting in a net increase in m6A abundance in the cortex and hippocampus of these mice. This discrepancy could simply be because of the use of different model systems (postmortem human AD brains vs an AD mouse model). However, given that we did not see a correlation between the alteration of METTL3 expression with Aβ load, future studies using Tau mouse models are warranted.

METTL3 and METTL14 form a stable dimer *in vivo*, and perturbation in one component leads to instability of the m6A-writer complex (Liu et al., 2014). Our analysis did not find alteration in the mRNA level of *METTL14* in AD despite a trend toward a reduced METTL14 protein level in the soluble fraction. However, the increase in the mRNA and protein levels of RBM15B, a key member of the MACOM that recruits METTL3/14 to mRNA (Patil et al., 2016), may reflect a compensatory response to the downregulation of the functional core m6A-methyltransferase complex in the brain. Overall, our study demonstrates dysregulation of key m6A regulatory enzymes in the human postmortem AD brain, implicating a perturbation in m6A signaling in the pathology of the disease, a possibility that warrants further investigation.

## Synthesis

Reviewing Editor: Aniko Korosi, University of Amsterdam Decisions are customarily a result of the Reviewing Editor and the peer reviewers coming together and discussing their recommendations until a consensus is reached. When revisions are invited, a fact-based synthesis statement explaining their decision and outlining what is needed to prepare a revision will be listed below. The following reviewer(s) agreed to reveal their identity: Fernando Peña-Ortega.

The authors have addressed all comment s satisfactorily
