# Supplementary material for: Altered Expression of the m6A Methyltransferase METTL3 in Alzheimer’s Disease
Source: eNeuro. 2020 Sep 8;7(5):ENEURO.0125-20.2020. doi: 10.1523/ENEURO.0125-20.2020 (PMC7540926; doi:10.1523/ENEURO.0125-20.2020)
Supplement: Extended Data Table 2-1 — Human brain samples from normal and Alzheimer’s disease patients analyzed in this study. Download Table 2-1, DOCX file. [file enu-eN-NWR-0125-20-s01.docx]

**Extended Table 2-1: Human brain samples from normal and Alzheimer’s disease patients analyzed in this study**

| Sample | Age | Sex | Tangle Stage | Plaque Stage | NPDx1 | ApoE | Soluble | Insoluble | DAB |
| --- | --- | --- | --- | --- | --- | --- | --- | --- | --- |
| Ctrl-1 | 94 | M | Stage 1 | None | Normal (MBC) | 3/3 | Y | Y |  |
| Ctrl-2 | 87 | F | Stage 2 | None | Normal (MBC) | 2/3 | Y | Y |  |
| Ctrl-3 | 86 | M | Stage 2 | None | Normal (MBC) | 3/3 | Y | Y | Y |
| Ctrl-4 | 95 | F | Stage 2 | None | Normal (MBC) | 3/3 | Y | Y | Y |
| Ctrl-5 | 96 | M | Stage 2 | None | Normal (MBC) | 2/3 | Y | Y | Y |
| Ctrl-6 | 86 | M | Stage 3 | Stage A | Normal (MBC) | 3/3 | Y | Y |  |
| Ctrl-7 | 91 | F | Stage 2 | Stage A | Normal (MBC) | 3/3 | Y |  | Y |
| Ctrl-8 | 91 | M | Stage 3 | Stage A | Normal (MBC) | 3/4 | Y | Y |  |
| Ctrl-9 | 91 | F | Stage 4 | None | Normal (MBC) | 2/3 | Y | Y |  |
| Ctrl-10 | 90 | M | Stage 3 | Stage A | Normal (MBC) | 3/4 | Y | Y |  |
| Ctrl-11 | 83 | F | Stage 4 | Stage A | Normal (MBC) | 3/3 | Y | Y |  |
| Ctrl-12 | 83 | M | Stage 5 | Stage C | Normal (MVC) | 3/4 | Y |  | Y |
| Ctrl-13 | 89 | F | Stage 3 | Stage B | Normal (MBC) | 3/3 | Y | Y |  |
| Ctrl-14 | 90 | F | Stage 3 | Stage B | Normal (MBC) | 3/3 | Y | Y | Y |
| Ctrl-15 | 93 | F | Stage 5 | Stage A | Normal (MBC) | 3/4 | Y | Y |  |
| Ctrl-16 | 97 | M | Stage 2 | Stage B | Normal (MBC) | 3/3 | Y | Y |  |
| Ctrl-17 | 91 | F | Stage 3 | Stage A | Normal (MBC) | 3/4 | Y | Y | Y |
| Ctrl-18 | 86 | F | Stage 3 | Stage C | Normal (MBC) | 3/3 | Y |  | Y |
| Ctrl-19 | 97 | M | Stage 4 | Stage B | Normal (MBC) | 3/4 |  | Y |  |
| AD-1 | 86 | M | Stage 5 | Stage C | AD | 3/3 | Y | Y | Y |
| AD-2 | 91 | F | Stage 6 | Stage C | AD | 3/3 | Y | Y | Y |
| AD-3 | 92 | M | Stage 5 | Stage C | AD | 3/3 | Y | Y |  |
| AD-4 | 95 | F | Stage 6 | Stage C | AD | 2/3 | Y | Y |  |
| AD-5 | 81 | M | Stage 6 | Stage C | AD | 3/4 | Y | Y | Y |
| AD-6 | 89 | F | Stage 6 | Stage C | AD | 3/4 | Y | Y |  |
| AD-7 | 90 | M | Stage 6 | Stage C | AD | 3/3 | Y | Y |  |
| AD-8 | 86 | M | Stage 6 | Stage C | AD | 3/4 | Y | Y |  |
| AD-9 | 92 | F | Stage 5 | Stage C | AD | 3/4 | Y | Y |  |
| AD-10 | 84 | M | Stage 5 | Stage B | AD | 3/4 | Y | Y |  |
| AD-11 | 93 | F | Stage 5 | Stage B | AD | 2/4 | Y | Y |  |
| AD-12 | 97 | M | Stage 5 | Stage B | AD | 3/3 | Y | Y |  |
| AD-13 | 93 | F | Stage 6 | Stage C | AD | 3/3 | Y | Y |  |
| AD-14 | 82 | F | Stage 6 | Stage C | AD | 3/3 | Y | Y | Y |
| AD-15 | 98 | F | Stage 6 | Stage C | AD | 3/3 | Y | Y |  |
| AD-16 | 88 | F | Stage 5 | Stage C | AD | 3/3 | Y | Y |  |
| AD-17 | 81 | M | Stage 6 | Stage C | AD | 3/3 | Y | Y |  |
| AD-18 | 96 | M | Stage 6 | Stage C | AD | 3/4 | Y |  |  |
| AD-19 | 95 | M | Stage 5 | None | AD | 3/4 | Y | Y |  |
| AD-20 | 89 | F | Stage 4 | Stage B | AD | 4/4 | Y | Y |  |
| AD-21 | 90 | F | Stage 5 | Stage B | AD | 3/3 | Y | Y |  |
| AD-22 | 90 | F | Stage 6 | Stage C | AD | 3/3 | Y | Y |  |
| AD-23 | 92 | F | Stage 5 | Stage C | AD | 3/4 | Y | Y |  |
| AD-24 | 75 | F | Stage 6 | Stage C | AD | 3/3 |  |  | Y |
| AD-25 | 81 | M | Stage 6 | Stage C | AD | 3/4 |  |  | Y |
| AD-26 | 78 | M | Stage 6 | Stage C | AD | 4/4 |  |  | Y |

NPDx1 (Neuropathological index); MBC, mild Braak changes; MVC, mild vascular changes; Y (yes) indicates samples that were analyzed in the indicated assay.
