# Supplementary figures and images for: Altered Expression of the m6A Methyltransferase METTL3 in Alzheimer’s Disease
Source: eNeuro. 2020 Sep 8;7(5):ENEURO.0125-20.2020. doi: 10.1523/ENEURO.0125-20.2020 (PMC7540926; doi:10.1523/ENEURO.0125-20.2020)

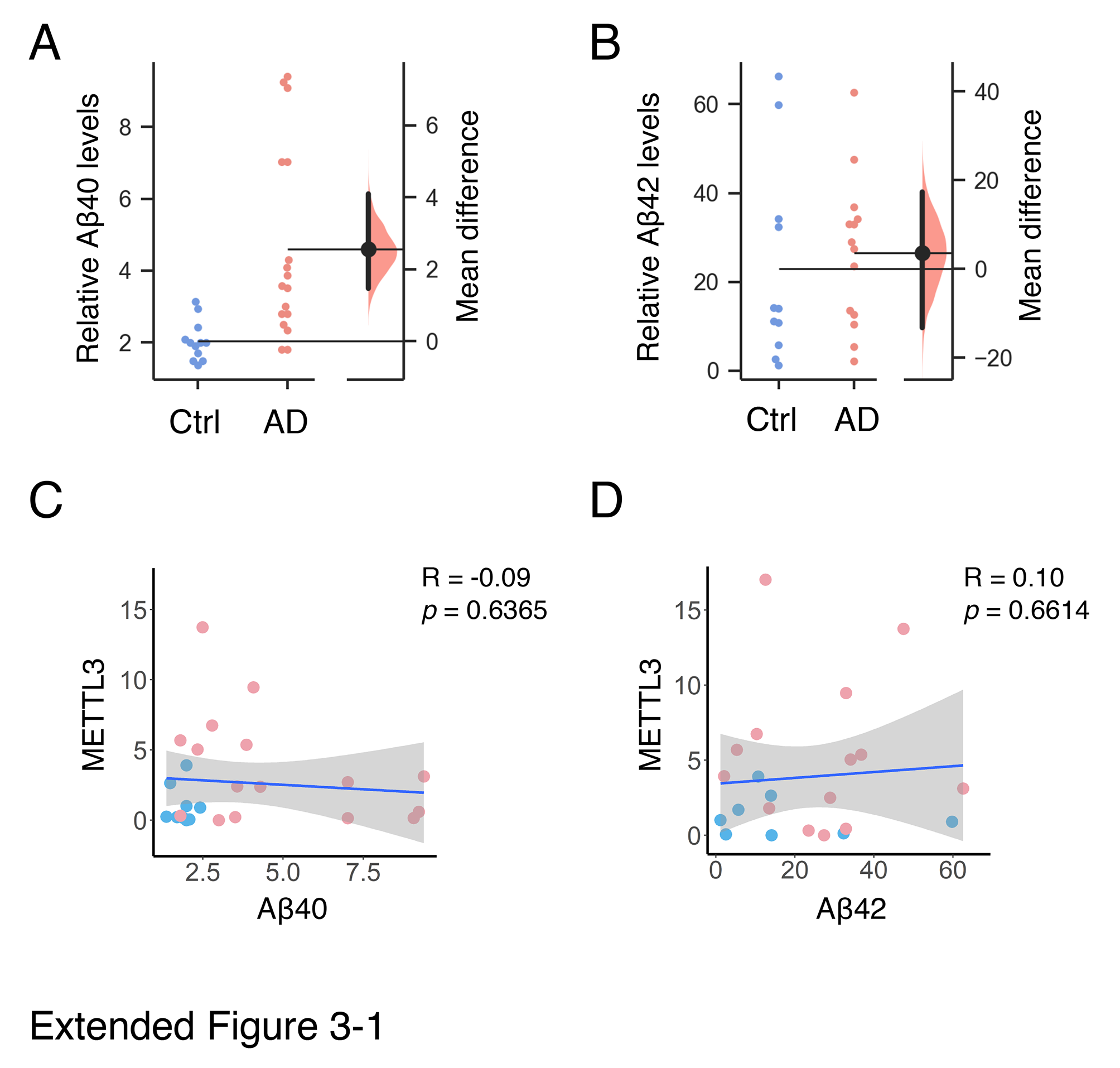

Supplement: Extended Data Figure 3-1 — The levels of Aβ40 or Aβ42 do not correlate with METTL3 accumulation in the insoluble fractions. Relative levels of Aβ40 (A) or Aβ42 peptides (B) of control (n = 11–12, blue dots) and AD (n = 14–17, pink dots) as measured by ELISA. Data are presented as estimation plots. Accumulation of METTL3 in the insoluble fraction of human hippocampal tissues does not correlate with Aβ40 (C) or Aβ42 (D) levels. Spearman’s correlation coefficients and p values are shown on each graph. Download Figure 3-1, TIF file. [file enu-eN-NWR-0125-20-s02.tif]
